# Investigation and Prediction of Human Interactome Based on Quantitative Features

**DOI:** 10.3389/fbioe.2020.00730

**Published:** 2020-07-17

**Authors:** Xiaoyong Pan, Tao Zeng, Yu-Hang Zhang, Lei Chen, Kaiyan Feng, Tao Huang, Yu-Dong Cai

**Affiliations:** ^1^School of Life Sciences, Shanghai University, Shanghai, China; ^2^Key Laboratory of System Control and Information Processing, Ministry of Education of China, Institute of Image Processing and Pattern Recognition, Shanghai Jiao Tong University, Shanghai, China; ^3^Key Laboratory of Systems Biology, Institute of Biochemistry and Cell Biology, Chinese Academy of Sciences, Shanghai, China; ^4^Shanghai Institute of Nutrition and Health, Shanghai Institutes for Biological Sciences, Chinese Academy of Sciences, Shanghai, China; ^5^College of Information Engineering, Shanghai Maritime University, Shanghai, China; ^6^Department of Computer Science, Guangdong AIB Polytechnic, Guangzhou, China

**Keywords:** decision tree, human interactome, prediction, protein–protein interaction, quantitative feature

## Abstract

Protein is one of the most significant components of all living creatures. All significant and essential biological structures and functions relies on proteins and their respective biological functions. However, proteins cannot perform their unique biological significance independently. They have to interact with each other to realize the complicated biological processes in all living creatures including human beings. In other words, proteins depend on interactions (protein-protein interactions) to realize their significant effects. Thus, the significance comparison and quantitative contribution of candidate PPI features must be determined urgently. According to previous studies, 258 physical and chemical characteristics of proteins have been reported and confirmed to definitively affect the interaction efficiency of the related proteins. Among such features, essential physiochemical features of proteins like stoichiometric balance, protein abundance, molecular weight and charge distribution have been validated to be quite significant and irreplaceable for protein-protein interactions (PPIs). Therefore, in this study, we, on one hand, presented a novel computational framework to identify the key factors affecting PPIs with Boruta feature selection (BFS), Monte Carlo feature selection (MCFS), incremental feature selection (IFS), and on the other hand, built a quantitative decision-rule system to evaluate the potential PPIs under real conditions with random forest (RF) and RIPPER algorithms, thereby supplying several new insights into the detailed biological mechanisms of complicated PPIs. The main datasets and codes can be downloaded at https://github.com/xypan1232/Mass-PPI.

## Introduction

Protein–protein interactions (PPI) are core biochemical events that directly execute biological functions in all living creatures (Qian et al., 2014; Wang et al., 2014). As the major executor of various biological processes, proteins rarely act alone, and protein interactions guarantee the continuity and controllability of ordinary biological processes (De Las Rivas and Fontanillo, 2010). On one hand, PPIs based on functional classification have multiple types, including signal transduction (Vinayagam et al., 2011), trans-membrane transport (Fairweather et al., 2015), cell metabolism (Gonzalez, 2012), and muscle contraction (Beqollari et al., 2015); these PPIs cover every detailed functional aspect in living cells. On the other hand, on the basis of chemical structure and stability, PPIs can be described as homo/hetero-oligomers, stable/transient interactions, and covalent/non-covalent interactions, thereby revealing the complicated chemical nature of common biochemical reactions that support protein interactions in all living cells (De Las Rivas and Fontanillo, 2010).

The complicated organization of PPIs can be clustered in multiple ways. Given the complexity and core regulatory role of protein interactions underlying biochemical processes in living cells, for a long time, many scientists have aimed to analyze and extract the key regulatory factors in the PPIs and describe their functional relationships and biological significance. According to previous studies, biochemical features of PPIs (e.g., protein concentration, protein binding ligands, presence of adaptors, and covalent modifications) have been recognized as candidate factors that may affect PPIs (Pan et al., 2010; Raj et al., 2013; Modell et al., 2016). However, most of such extracted features are ambiguous qualitative characteristics. These features may be directly or indirectly related to PPIs, but whether PPIs with optimal biological features may be determined in certain cell types is difficult. These features are not detailed differentiating indicators for the occurrence possibility of PPIs, rather than existence. Therefore, accurate and quantitative/semi-quantitative characteristics of PPIs must be identified through continuous studies and exploration.

In recent years, with the development of mass spectrometry and related analysis techniques, various omics features have been presented to describe the characteristics of PPIs and have been applied to evaluate the possibility and certain biological functions of cell-specific PPIs. In 2015, using high-throughput affinity-purification mass spectrometry, Huttlin et al. (2015) built a PPI network (BioPlex) and extracted various functional characteristics describing PPIs, thus providing us with a blueprint of quantitative human interactome in all living cells. In the same year, another study presented by Wan et al. focused on the macromolecular complexes' contribution to PPIs; these authors extracted the co-complex interactions using an integrative approach (Wan et al., 2015), thereby revealing the fundamental mechanistic significance of reconstructed interactomes. This study also extracted a group of parameters/features that can be used for a detailed quantitative description of PPI. In 2015, another study by Hein et al. (2015) further proposed nine features, such as NWD, Z, and Plate Z scores, which may quantitatively describe PPIs. Combining the datasets of the three studies, a systemic analysis of all reported human protein complexes based on mass spectrometry techniques has been recently presented (Drew et al., 2017). Such study summarized the identified features associated with PPIs (i.e., PPI features) and built a global map of all reported human protein complexes. It provided us with a database, namely hu.MAP (http://proteincomplexes.org/), as a new resource of a follow-up study on the core physical and pathological functions of human PPIs in normal and disease cells. Such features captured the specificity of real PPIs and were screened out by three independent studies (Hein et al., 2015; Huttlin et al., 2015; Wan et al., 2015). According to such studies (Hein et al., 2015; Huttlin et al., 2015; Wan et al., 2015), all candidate features are validated by large scale mass spectrometry and have been identified to contribute to the regulation and description of certain PPIs.

However, the original and combination studies of three datasets have not identified the key factors that may contribute to and appropriately describe the occurrence possibility of PPIs. Previous studies have merely identified and summarized potential PPI features, but the significance comparison and quantitative contribution of candidate PPI features remain to be identified. Thus, in this study, the PPI data obtained from multiple mass spectrometry experiments (Drew et al., 2017) is summarized by our newly presented decision tree-centered computational framework. Such PPI data contained one training dataset and one testing dataset, each of which consisted of proteins that can interact with each other, namely positive PPIs, and proteins that cannot interact with each other, namely negative PPIs. The core parameters of PPI features that may describe and judge the possibility of potential PPIs are accurately identified. The decision tree-based model with extracted core PPI features yielded better performance than the models with other classification algorithms, including nearest neighbor algorithm (NNA) (Cover and Hart, 1967) and recurrent neural network (RNN). Furthermore, a quantitative decision-rule system based on PPI features is built to supply several new insights into the detailed biological mechanisms of complicated PPIs. These quantified outcomes not only reveal the core regulatory factors in PPIs but also provide a new computational tool for investigating and predicting the potential of PPIs under different physical and pathological conditions.

## Materials and Methods

### Datasets

The training and testing human PPI datasets were obtained from Drew et al. (2017) (http://proteincomplexes.org/download). The training dataset has 68,651 PPIs, in which 9,318 are actual positive PPIs (i.e., proteins that can interact with each other), and 59,333 are negative PPIs (i.e., proteins that cannot interact with each other). These PPIs cover 1,253 proteins. The testing dataset has 77,884 PPIs, in which 4,579 are actual positive PPIs, and 73,305 are negative PPIs. One thousand one hundred thirty-two proteins occur in the testing dataset, where 606 are also used in the training dataset. Each PPI was encoded with 258 features, which were downloaded from Drew et al. (2017) too. They were defined in three previous studies (Hein et al., 2015; Huttlin et al., 2015; Wan et al., 2015) and represented various biological characteristics of PPI. Only human proteins were included and the PPIs were literature-curated.

To describe the PPIs, we summarized the features described in three publications: Wan et al. (2015), BioPlex (Huttlin et al., 2015), and Hein et al. (2015). There were 241 features from Wan et al. (2015), 11 features from BioPlex (Huttlin et al., 2015) and 6 features from Hein et al. (2015). These co-fractionation and physiochemical features described all the properties that may affect the potential interactions between the target protein either partially or as an entity. These features had been refined with mass spectrum results (Hein et al., 2015). The redundant and unimportant features had been removed to establish an effective framework for PPIs description using co-fractionation and physiochemical features. For instance, there is a specific feature named as spatiotemporal overlap (Hein et al., 2015), describing the temporal spatial interactions between two participators of PPIs. Interactions with either too high spatiotemporal overlap or too low overlap may indicate the interaction will not actually happen (Hein et al., 2015). All the features used in this study are summarized from existed datasets and derived from experimental results.

### Feature Selection

In this study, a three-stage feature selection scheme was designed to identify important features for characterizing PPIs. In the first stage, all features were analyzed by the Boruta feature selection (BFS) (Kursa and Rudnicki, 2010) method, excluding irrelated features; then, the rest features were analyzed by the Monte Carlo feature selection (MCFS) (Draminski et al., 2008) method, producing a feature list; finally, the feature list was adopted in the incremental feature selection (IFS) (Liu and Setiono, 1998) method, incorporating a supervised classifier, to extract optimal features and build an optimal classifier.

#### Boruta Feature Selection Method

BFS method (Kursa and Rudnicki, 2010) is a wrapper method for selecting relevant features, which is based on random forest (RF) (Breiman, 2001). It evaluates feature importance by comparing with randomized features. Such method is different from most of the other wrapper feature selection methods that achieve a minimal error for a supervised classifier on a small subset of features, BFS selects all features either strongly or weakly relevant to the outcome variable.

The core idea of BFS is that it creates a shuffled version of original features, then uses a RF classifier to measure the importance score of the combined shuffled and original features. Only those features with importance score higher than that of the randomized features are selected. These selected features are considered significantly relevant to target variables. The difference between RF importance score and BFS importance score is that the statistical significance of the variable importance is introduced. Random permutation procedure is repeated to get statistically robust important features. BFS proceeds as follows by repeating multiple iterations:

1. Add randomness to the given dataset by shuffling original features.
2. Combine the shuffled dataset and original dataset.
3. Train a RF classifier on the combined dataset and evaluate the importance of each feature.
4. Calculate Z-scores of both original and shuffled features. The Z-scores of individual features are calculated as mean of importance scores divided by the standard error. For each real feature, evaluate whether it has a higher Z-score than the maximum of its shuffled feature. If yes, this feature is tagged as important, otherwise unimportant.
5. Finally, the algorithm stops until one of the two following condition is satisfied: (I) All features are either tagged “unimportant” or “important”; (II) Reach a predefined number of iterations.

In this study, we used the python implementation of BFS from https://github.com/scikit-learn-contrib/boruta_py, and the defaulted parameters are used.

#### Monte Carlo Feature Selection Method

As mentioned in section Boruta Feature Selection Method, features selected by BFS method are highly related to target variables. These features are further analyzed by the MCFS method (Draminski et al., 2008). MCFS is a powerful and widely used feature selection method (Chen L. et al., 2018a, 2019b; Pan et al., 2018, 2019; Wang et al., 2018), which consists of multiple decision trees, and constructs multiple bootstrap sets and randomly selects feature subsets. For each feature subset, new training samples are re-represented by using the features in this subset, and *M* decision trees are grown by using the bootstrap sets sampled from the new training samples. This process is repeated *T* times, thereby resulting in *M* × *T* trees. A relative importance (RI) score is calculated in accordance with the involvement of a feature in constructing *M* × *T* trees. Its equation is as follow:

(1) $$\begin{array}{l} RI_{g}=\overset{MT}{\underset{\tau =1}{\sum }}{\left(wAcc\right)}^{u}IG\left(n_{g}\left(\tau \right)\right){\left(\frac{no.in\;n_{g}\left(\tau \right)}{no.in\;\tau }\right)}^{v}, \end{array}$$

where *g* stands for a feature, wAcc denotes the weighted accuracy of the decision tree τ, *n*_*g*_(τ) represents the node involving *g* in τ, *IG*(*n*_*g*_(τ)) represents the information gain of *n*_*g*_(τ), *no*.*in τ* and *no*.*in n*_*g*_(τ) denotes the number of samples in decision tree τ and node *n*_*g*_(τ), respectively. *u* and *v* are weighting factors. Evidently, a high RI score indicates that one feature will be more frequently involved in learning these decision trees. Thus, this feature will have ranked relevance in characterizing PPIs. Based on the RI scores of features, a feature list, denoted as *F* = [*f*_1_, *f*_2_, …, *f*_*N*_], can be built by the decreasing order of features' RI scores.

The MCFS program was downloaded from http://www.ipipan.eu/staff/m.draminski/files/dmLab_2.1.1.zip. We used the default parameters to execute such program, where *u* and *v* were set to 1, *M* and *T* were 2,000 and 5, respectively.

#### Incremental Feature Selection Method

A feature list can be generated according to the results of MCFS method, based on which incremental feature selection (IFS) (Liu and Setiono, 1998; Li et al., 2015, 2016, 2019; Chen et al., 2017b; Chen L. et al., 2018b, 2019a; Wang and Huang, 2018; Zhang et al., 2018), combining with a supervised classifier (i.e., RF), is adopted to further detect discriminative features for indicating PPIs. A series of feature subsets is generated from the ranked features *F* from the MCFS. The first feature subset has feature *f*_1_, the second feature subset has features [*f*_1_, *f*_2_], and so on. RF is run to test these feature subsets with 10-fold cross validation. Finally, an RF classifier with the optimal classification performance is generated, such classifier was termed as the optimal classifier. And the features in the corresponding feature subset are called optimal features (i.e., PPI features).

## Smote

It is easy to see that the negative PPIs were much more than positive PPIs in both training and testing datasets. In detail, in the training dataset, negative PPIs were about 6.37 times as many as positive PPIs, while such proportion was about 16 for the testing dataset. Thus, the investigated datasets were greatly imbalanced. For such type of dataset, it is not easy to build a perfect classifier. In this study, we employed Synthetic Minority Over-sampling Technique (SMOTE) (Chawla et al., 2002) to tackle such datasets.

SMOTE is a classic and widely used oversampling method. It generates predefined numbers of samples and pours them into the minority class. In detail, it first randomly selects a sample in one minority class, say *x*. Then, find *k* samples in such class, which have smallest distances to *x*. Randomly select a sample from these *k* samples, say *y*, and generate a new sample *z*, which is the linear combination of *x* and *y*. The generated new sample *z* is put into the minority class. Above procedures execute multiple times until predefined number of new samples have been produced.

In this study, we directly adopted the tool “SMOTE” in Weka (Version 3.6) (Witten and Frank, 2005), which implement above-mentioned SMOTE. For the training dataset, we used “SMOTE” generated lots of new samples and termed them as positive PPIs. Finally, the numbers of positive and negative PPIs were almost equal. We used the default value of parameter *k*, which was 3. As suggested in Blagus and Lusa (2013), feature selection should be performed before using SMOTE. Thus, in this study, the SMOTE was only adopted in IFS method. Samples yielded by SMOTE were not used in the BFS and MCFS methods.

### Classifier

In IFS method, supervised classifiers are indispensable. Here, two classic classifiers were adopted. They were RF (Breiman, 2001) and RIPPER algorithm (Cohen, 1995). The first one was to build an efficient classifier. However, it cannot bring lots of information to uncover the essential differences between positive and negative PPIs. Thus, we further employed the second classifier, RIPPER algorithm, which is a rule learning algorithm. It can provide several rules to clearly display the classification procedures and differences between positive and negative PPIs.

#### Random Forest

As a supervised classifier, RF consists of multiple decision trees, and each decision tree is grown from a bootstrap set and a randomly selected feature subset. We assume a training set with *N* samples and *M* features. For each decision tree, the same number of samples is first randomly selected from the original training set with replacement and a feature subset with *m* features (*m* < < *M*) is also randomly constructed. Each tree is grown from these selected samples with the selected feature subset. This process is repeated *T* times, and *T* decision trees comprising the RF are yielded. RF has much fewer parameters to tune; thus, this technique is extensively used in many biological problems with favorable performance (Pan et al., 2010, 2014; Zhao et al., 2018, 2019; Zhang et al., 2019). The RF classifier implemented by a tool “RandomForest” in Weka (Witten and Frank, 2005) software is used. Clearly, the number of decision trees is an important parameter of RF. Here, we tried four values: 10, 20, 50, and 100.

#### Repeated Incremental Pruning to Produce Error Reduction Algorithm

**Figure 1:**
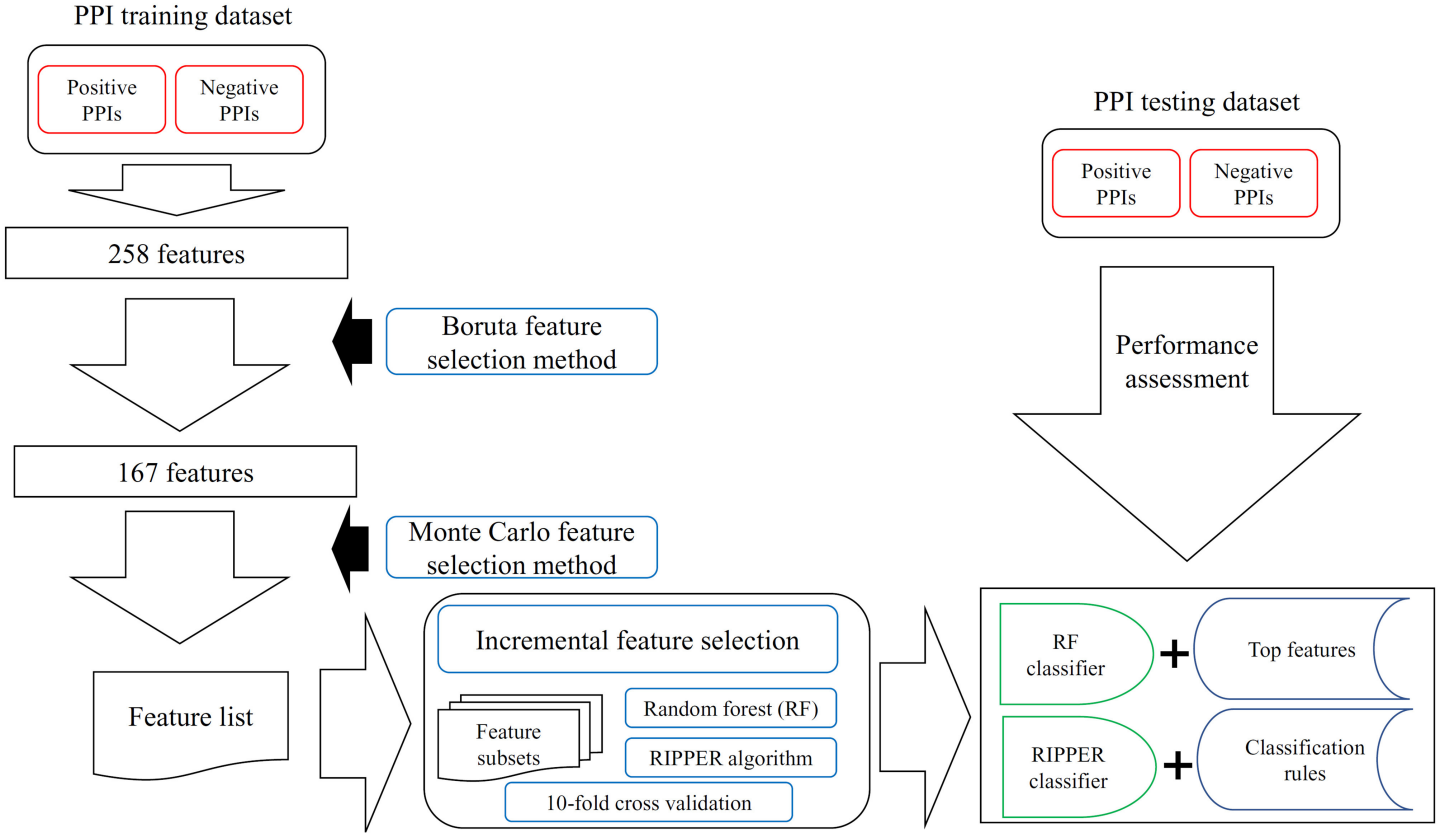
The entire procedures to analyze PPI features with a three-stage feature selection scheme. The PPI samples comprise one training dataset and one testing dataset. For the training samples, they were represented by 258 features, which are processed by Boruta feature selection method. One hundred sixty-seven relevant features remain, which are further analyzed by the Monte Carlo feature selection method. A feature list is produced. The incremental feature selection uses such feature list to construct several feature subsets. On each subset, one random forest (RF) classifier and one RIPPER classifier are constructed, which are assessed by 10-fold cross-validation. With RF, the best RF classifier and top features are obtained; whereas with RIPPER, the best RIPPER classifier together with its rules is generated. The best RF and RIPPER classifiers are further evaluated on the testing dataset.

RIPPER algorithm (Cohen, 1995) is a classic rough set based rule learning algorithm. In fact, it is a generalized version of the Incremental Reduced Error Pruning (IREP) algorithm (Johannes and Widmer, 1994). The procedures of rule learning with RIPPER can be found in our previous study (Figure 1; Wang et al., 2018). Rules generated by RIPPER algorithm are represented by IF-THEN clauses. For example, IF (Feature 1 ≥2.333 and Feature 2 ≤ 1.234) THEN Positive PPI. Likewise, RIPPER algorithm is also implemented by a tool “JRip” in Weka (Witten and Frank, 2005). We directly used it and executed it with its default parameters.

### Performance Measurement

The performance of the classifiers is evaluated using 10-fold cross validation. Several evaluation metrics, such as sensitivity (SN), specificity (SP), two types of accuracy (ACC1 and ACC2), Matthew correlation coefficient (MCC) (Matthews, 1975; Chen et al., 2017a; Chen Z. et al., 2018, 2019; Li et al., 2018; Song et al., 2018; Cui and Chen, 2019), recall, precision, and F-measure are calculated and formulated as follows:

(2) $$\begin{array}{l} SN=\frac{TP}{TP+FN}, \end{array}$$

(3) $$\begin{array}{l} SP=\frac{TN}{TN+FP}, \end{array}$$

(4) $$\begin{array}{l} ACC1=\frac{TP+TN}{TP+TN+FP+FN}, \end{array}$$

(5) $$\begin{array}{l} ACC2=\left(SN+SP\right)/2 \end{array}$$

(6) $$\begin{array}{l} MCC=\frac{TP\times TN-FP\times FN}{\sqrt{\left(TP+FP\right)\left(TP+FN\right)\left(TN+FP\right)\left(TN+FN\right)}}, \end{array}$$

(7) $$\begin{array}{l} Recall=\frac{TP}{TP+FN}, \end{array}$$

(8) $$\begin{array}{l} Precision=\frac{TP}{TP+FP}, \end{array}$$

(9) $$\begin{array}{l} F-measure=\frac{2\times Precision\times Recall}{Precision+Recall}, \end{array}$$

where TP/TN are the numbers of true positives/negatives, and FP/FN are the numbers of false positives/negatives. Clearly, ACC1, ACC2, MCC, and F-measure can fully evaluate the performance of a classifier. This study selected F-measure as the key measurement.

In addition to above-mentioned measurements, we also employed ROC and PR curves to fully evaluate the performance of different classifiers. The areas under these two curves are also important measurements to assess classifiers. They were called AUROC and AUPR, respectively, in this study.

## Results

In this study, the prior extracted 258 features were analyzed by a three-stage feature selection scheme. The entire procedures are illustrated in Figure 1.

### Analysis of the Identity Between PPIs in the Training and Testing Datasets

**Figure 2:**
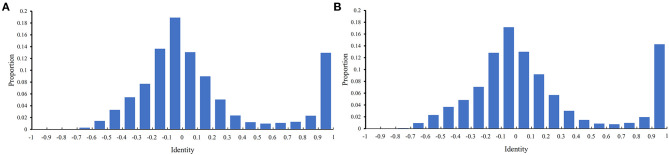
Distribution of identities between PPIs in two datasets. The identify of two PPIs is defined as the direction cosine of their feature vectors. **(A)** Distribution on the training dataset; **(B)** Distribution on the testing dataset.

Before performing the feature selection scheme, it is necessary to count the identity between PPIs in the training and testing datasets because PPIs with high identities will make the classification easily. Here, the identity between two PPIs was defined as the direction cosine of their 258-D feature vectors. We used 0.1 as the step to count the distribution of the obtained identities on the training and testing datasets, which is shown in Figure 2. It can be observed that the training and testing datasets gave the similar distribution on identities. The interval [−0.1,0] contained the most identities and between −1 and 0.6, the distribution was quite similar to the normal distribution. It is also surprised that several identities were with high values (interval [0.9, 1]). However, more than 80% identities were <0.5, indicating that most PPIs were with low identities. The investigation on such datasets was quite reliable.

### Results of Boruta Feature Selection (BFS) Method

In the training dataset, all PPIs were represented by 258 features. These features were analyzed by BFS method. As a result, 167 features were selected, as listed in Table S1.

### Results of Monte Carlo Feature Selection (MCFS) Method

According to the three-stage feature selection scheme, remaining 167 features were analyzed by the powerful MCFS method. Each feature was assigned a RI score, which is also provided in Table S1. Accordingly, a feature list F was built, in which features were sorted by the decreasing order of their RI scores. This list is available in Table S1.

### Results of Incremental Feature Selection (IFS) With Random Forest (RF)

**Figure 3:**
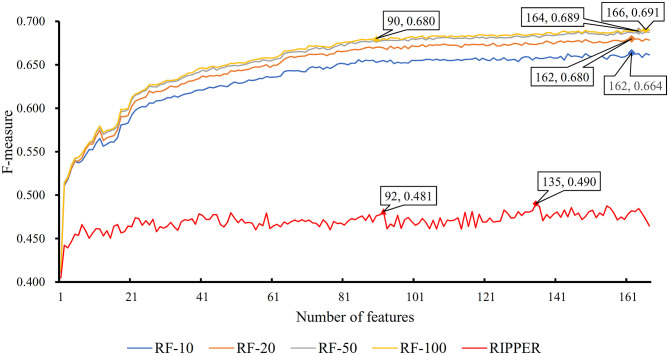
IFS curves based on the IFS method with RF and RIPPER. The X-axis shows the number of features, and Y-axis shows the F-measure values. The numbers following RF indicate the number of decision trees of RF.

**Figure 4:**
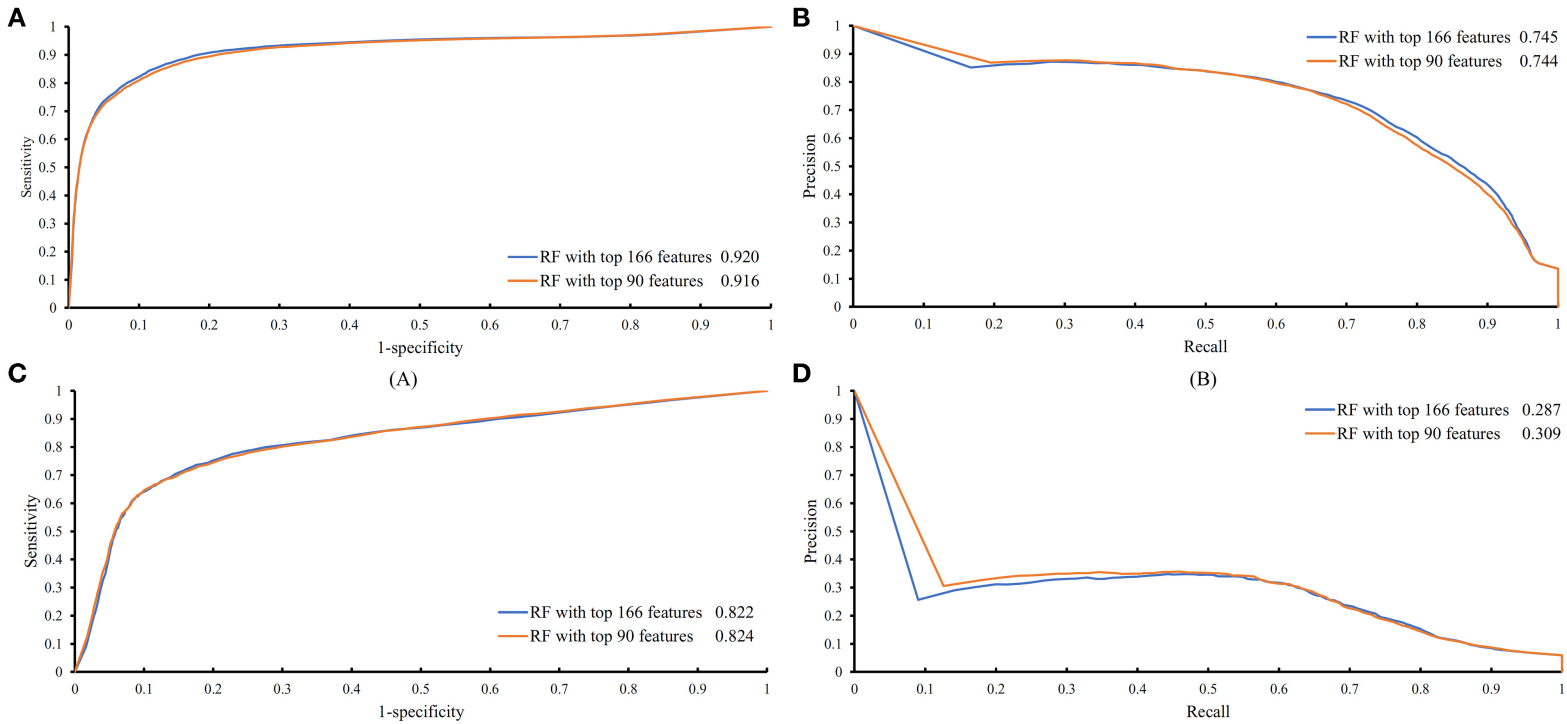
ROC and PR curves of the RF classifiers with top 166 and 90 features on the training and testing datasets. **(A)** ROC curves of two RF classifiers on the training dataset; **(B)** PR curves of two RF classifiers on the training dataset; **(C)** ROC curves of two RF classifiers on the testing dataset; **(D)** PR curves of two RF classifiers on the testing dataset.

The feature list only told us the importance of each feature. To extract optimal features for RF, IFS method was employed. For each feature subset constructed from *F*, RF classifiers with different number of decision trees (10, 20, 50, and 100) were built on the training dataset and evaluated through 10-fold cross validation. The results are provided in Tables S2–S5. To clearly display these RF classifiers on different feature subsets, four IFS-curves are plotted in Figure 3. It can be seen that the optimal F-measure value was 0.691 when the top 166 features in *F* were used and the number of decision trees was 100. Accordingly, the RF classifier containing 100 decision trees was built on the training dataset, in which PPIs were represented by top 166 features in *F*. Such classifier was called the optimal RF classifier. Other measurements yielded by such RF classifier are listed in Table 1. The SN, SP, ACC1, ACC2, MCC, and Precision were 0.794, 0.921, 0.903, 0.858, 0.642, and 0.611, respectively, suggesting the good performance of such classifier. Besides, we also used ROC curve and PR curve to evaluate the performance of such RF classifier, which are shown in Figures 4A,B. The AUROC and AUPR was 0.920 and 0.745, respectively.

**Table 1:** Performance of the RF and RIPPER classifiers on the training dataset evaluated by 10-fold cross-validation.

**Classifier**	**Number of features**	**SN**	**SP**	**ACC1**	**ACC2**	**MCC**	**Precision**	**F-measure**
RF	166	0.794	0.921	0.903	0.858	0.642	0.611	0.691
	90	0.786	0.918	0.900	0.852	0.630	0.600	0.680
RIPPER	135	0.701	0.818	0.802	0.760	0.409	0.377	0.490
	92	0.689	0.815	0.798	0.752	0.397	0.370	0.481
NNA	101	0.851	0.881	0.877	0.866	0.607	0.529	0.652
RNN	133	0.824	0.890	0.881	0.857	0.605	0.542	0.654

**Figure 5:**
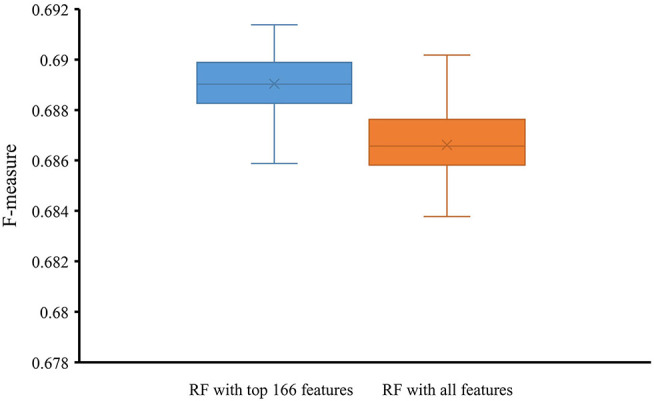
Box plot to show F-measures yielded by RF classifiers with top 166 features and all features using 50 10-fold cross-validation. The F-measures obtained by RF classifier with top 166 features are evidently higher than those of the RF classifier with all features.

To indicate the improvement of the RF with top 166 features, we conducted 10-fold cross-validation on this classifier 50 times. Also, the RF classifier with all 258 features were evaluated by 10-fold cross-validation 50 times. Obtained F-measures are shown in Figure 5, from which we can see that F-measures yielded by the RF classifier with top 166 features were evidently higher than those produced by the RF classifier with all features. To confirm this result, a paired sample *t*-test was conducted, yielding the *p*-value of 1.309E-15, suggesting that the performance of the RF classifier was improved with statistical significance.

Above-constructed RF classifier was also applied to the testing dataset. The predicted results are listed in Table 2, from which we can see that the F-measure was 0.371. Its SN, SP, ACC1, ACC2, MCC and Precision were 0.674, 0.877, 0.865, 0.776, 0.358, and 0.256, respectively. The ROC and PR curves of the constructed RF classifier on the testing dataset are shown in Figures 4C,D. The AUROC and AUPR was 0.822 and 0.287, respectively. Although they were lower than those on training dataset, the ACC1 was still over 0.850.

**Table 2:** Performance of the RF and RIPPER classifiers on the testing dataset.

**Classifier**	**Number of features**	**SN**	**SP**	**ACC1**	**ACC2**	**MCC**	**Precision**	**F-measure**
RF	166	0.674	0.877	0.865	0.776	0.358	0.256	0.371
	90	0.677	0.874	0.863	0.776	0.356	0.252	0.367
RIPPER	135	0.797	0.826	0.825	0.812	0.360	0.223	0.348
	92	0.800	0.822	0.821	0.811	0.357	0.219	0.344

As mentioned above, for RF with 100 decision trees, when top 166 features in *F* was used, it provided the best F-measure. However, after carefully checking the IFS results (Table S2), when top 90 features were used, RF can yield the F-measure of 0.680, which was a little lower than that yielded by the optimal RF classifier. Considering the efficiency of classifiers, we suggested the RF constructed on top 90 features as the proposed classifier. The detailed performance of this classifier, evaluated by 10-fold cross-validation, is provided in Table 1 and the ROC and PR curves are shown in Figures 4A,B. Clearly, the performance of this classifier was almost equal to that of the optimal RF classifier. Besides, the proposed classifier was also performed on the testing dataset, obtained measurements are listed in Table 2 and ROC and PR curves are shown in Figures 4C,D. Clearly, they all approximated to those of the optimal RF classifier. All of these indicated that the proposed RF classifier can provide similar results, however, it had high efficiency because much less features were involved.

### Comparison of IFS With NNA and RNN

**Figure 6:**
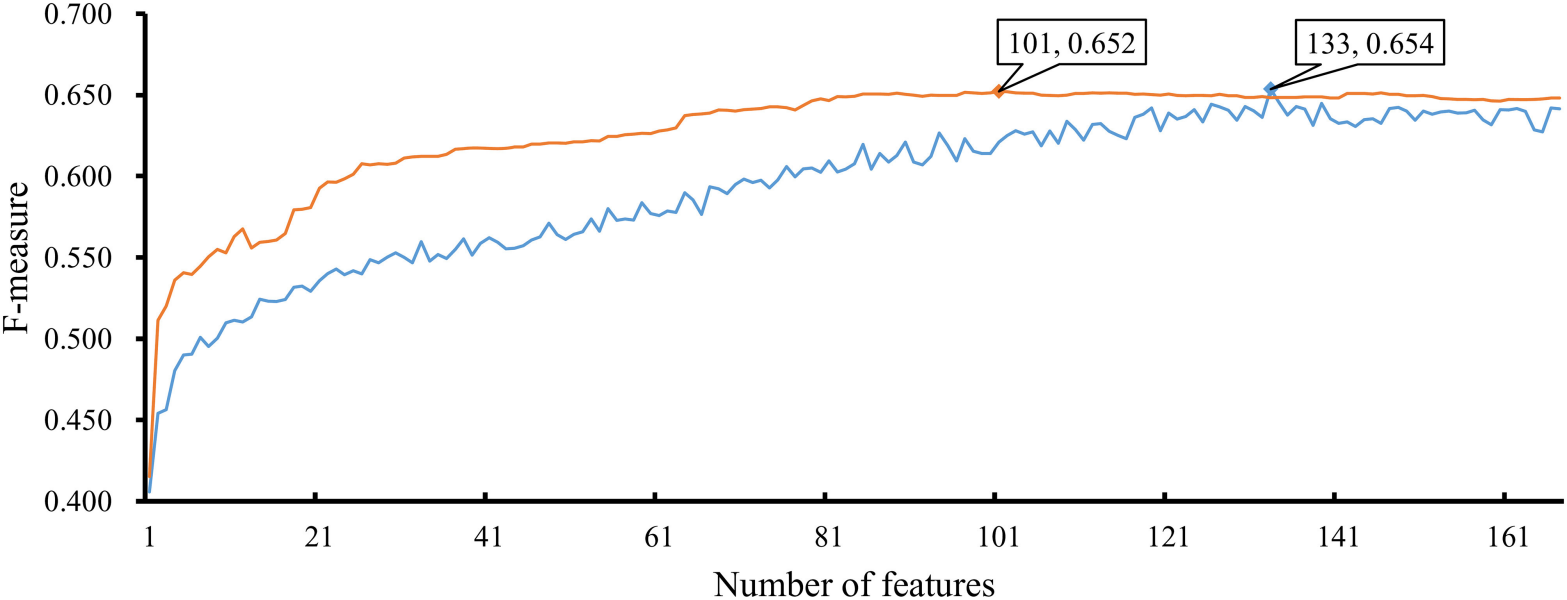
IFS curves based on the IFS method with NNA and RNN. The X-axis shows the number of features, and Y-axis shows the F-measure values. The orange curve and blue curve were NNA and RNN, respectively.

As mentioned above, the optimal RF classifier gave good performance. However, is the RF a proper choice? In fact, we also tried other two classification algorithms: NNA and RNN. NNA is a classic and simple classification algorithm, which makes prediction for a given sample according to its nearest neighbor, while RNN is a kind of neural network with loop inside for sequential data. For each of these two algorithms, an IFS procedure was performed on the training dataset. Two IFS curves were obtained, as shown in Figure 6. The highest F-measure for NNA was 0.652 when top 101 features in F were used. For RNN, the highest F-measure was 0.654 when top 133 features were adopted. These F-measure values were all lower than that of the optimal RF classifier. The detailed performance of the best NNA and RNN classifiers is listed in Table 1. It can be observed that the optimal RF classifier produced higher values on most measurements, suggesting that RF is a more proper choice than NNA and RNN.

### Results of IFS With RIPPER

In section Results of incremental feature selection (IFS) with random forest (RF), a RF classifier was built to identify PPIs. However, it is a black box. It is difficult to capture the classification principle. Thus, it provided limited biology insights for understanding PPIs. In view of this, we further employed a rule learning method, RIPPER algorithm, trying to partly uncover the differences between positive and negative PPIs.

Like RF, the RIPPER algorithm was also employed in the IFS method. The performance of the RIPPER algorithm on different feature subsets is available in Table S6. Also, an IFS-curve was plotted, as shown in Figure 3. The highest F-measure was 0.490 when top 135 features were used. Thus, the RIPPER classifier based on top 135 features was called the optimal RIPPER classifier. The detailed performance of such classifier, evaluated by 10-fold cross-validation, was provided in Table 1. Clearly, it was much inferior to the optimal RF classifier. In addition, the optimal RIPPER classifier was also executed on the testing dataset. The predicted results were listed in Table 2. The F-measure was 0.348, which was also much lower than that on the training dataset. Compared with the performance of the optimal RF classifier on the testing dataset, the performance of the optimal RIPPER classifier was only a little lower.

Likewise, the RIPPER classifier can yield the F-measure 0.481 on the training dataset when top 92 features were used after checking the predicted results listed in Table S6. It is a little lower than that generated by the optimal RIPPER classifier. Considering the efficiency of classifiers, we termed the RIPPER classifier with top 92 features as the proposed RIPPER classifier. The detailed performance of such classifier on the training dataset is listed in Table 1. All measurements were almost equal to those yielded by the optimal RIPPER classifier. Furthermore, the proposed RIPPER classifier was executed on the testing dataset. Predicted results are listed in Table 2. Obviously, the performances of the optimal and proposed classifiers were at the same level.

As mentioned above, the proposed RIPPER classifier adopted top 92 features to represent PPIs. Six rules were produced by the RIPPER algorithm when such algorithm was applied on all PPIs in the training dataset, which are listed in Table 3. These rules would be discussed in section Analysis of Optimal PPI Rules.

**Table 3:** Classification rules for predicting protein-protein interactions.

**Rules**	**Criteria**	**Positive/Negative**
Rule1	(neg_ln_pval ≤3.622) and (hein_neg_ln_pval ≤3.328)	Negative (non-interaction) PPI
Rule2	(hein_neg_ln_pval ≤6.955) and (Hs_G166_1104_pq_euc ≤0) and (neg_ln_pval ≤3.994)	Negative (non-interaction) PPI
Rule3	(hein_neg_ln_pval ≤6.960) and (neg_ln_pval ≤5.780) and (Hs_G166_1104_pq_euc ≤0) and (pair_count ≥2)	Negative (non-interaction) PPI
Rule4	(hein_neg_ln_pval ≤3.033) and (Hs_G166_1104_pq_euc ≤0) and (pair_count ≤3) and (neg_ln_pval ≤7.272)	Negative (non-interaction) PPI
Rule5	(hein_neg_ln_pval ≤0) and (Hs_G166_1104_pq_euc ≤0) and (pair_count ≤3) and (neg_ln_pval ≤8.611)	Negative (non-interaction) PPI
Rule6	Other conditions	Positive (interaction) PPI

## Discussion

All PPI-associated features have been summarized in the three previously described datasets (Hein et al., 2015; Huttlin et al., 2015; Wan et al., 2015). In this study, we deeply analyzed these features. Based on some key features, a RF classifier was constructed and some classification rules were built. This section gave detailed analysis on some top features and classification rules. Several top features and all rules were supported by recent publications (Mitterhuber, 2008; Swiatkowska et al., 2008; Levin et al., 2013; Pinton et al., 2015).

### Analysis of Optimal PPI Features

**Figure 7:**
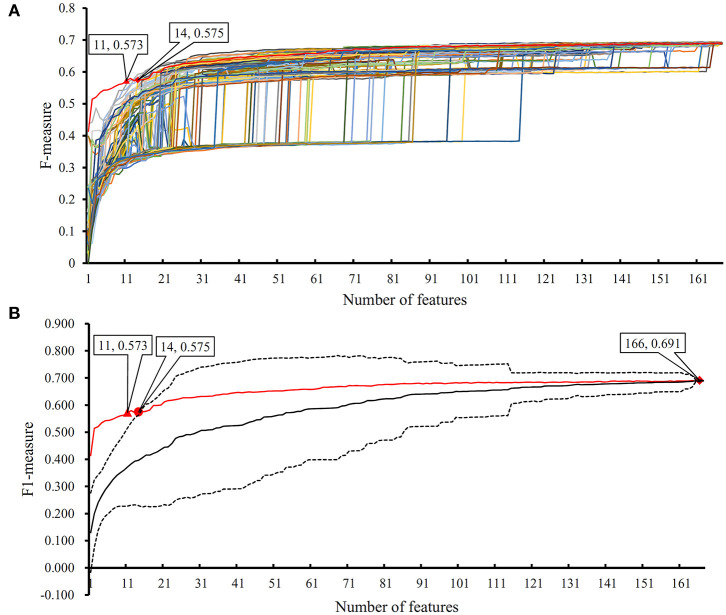
The results of the IFS method with RF based on 100 randomly produced feature lists. **(A)** IFS curves on the actual feature list and 100 randomly produced feature lists; **(B)** the statistical analysis based on the results of randomly produced feature lists. The black curve indicates the average performance of RF on randomly produced feature lists. The red curve is the IFS curve of the actual feature list. Two dotted curves indicate the upper and low critical value on 95% confidence interval.

In the proposed RF classifier, top 90 features were used to represent PPIs. However, it is impossible to analyze them one by one due to our limited human resources. In fact, among these 90 features, some were more important than others. We did the following test to extract most important features. Firstly, 100 feature lists were randomly built, in which 167 features were randomly sorted. According to each feature list, we did the IFS method with RF (consisting of 100 decision trees) procedures. As a result, 100 IFS-curves were plotted, as shown in Figure 7A, in which the IFS-curve produced on the actual feature list *F* is also listed. It can be observed that when the number of used features was small, the F-measure on the actual feature list *F* was much higher than those on the randomly generated feature lists, indicating that some top features in *F* were related to identify PPIs with high statistical significance. Thus, given a feature number, we counted the mean values of 100 F-measures that were produced on 100 randomly generated feature lists. Then, an IFS-curve was plotted, as shown in Figure 7B. Furthermore, we also counted the critical values on 95% confidence interval for each feature number and plotted two IFS-curves on them, as shown in Figure 7B. It can be observed that top 14 features in *F* can produce the F-measure that was higher than the upper critical value on 95% confidence interval, indicating that these 14 features were highly related to identify PPIs. Furthermore, top 11 features in *F* can yield the F-measure that was higher than the upper critical value on 99% confidence interval. In the following text, we extensively analyzed top 14 features in *F*.

The first four features are “hein_neg_In_pval,” “neg_In_pval,” “hein_pair_count,” and “pair count,” reflecting the regulatory contribution of protein stoichiometric and abundant features. In accordance with a reference dataset presented by Hein et al. (2015), these features were confirmed to participate in and may affect the content of interactome. According to the stoichiometric and abundant levels, a stable protein complex denotes a probable involvement of such protein complex in functional PPIs. Two detailed features, namely, stoichiometric balance and protein abundance, might generally evaluate the stability of a protein complex and participate in describing PPIs. The stable PPIs formed by stoichiometric balance might be further shaped by the abundance of each protein that participates in such interactions.

To clearly describe what are stoichiometric and abundant features, here, we took two typical PPIs as effective examples to confirm the potential contribution of such two features on the PPIs.

Firstly, we took the effective PPIs during cell adhesion regulation and functioning as an example. The adhesive properties of endothelial cells have been confirmed to be regulated by various proteins and their potential interactions (Swiatkowska et al., 2008). According to recent publications (Swiatkowska et al., 2008; Levin et al., 2013), actually among such interactions, the abundance and stoichiometric balance of disulfide isomerases and integrin may directly affect their PPIs and further interfere endothelial cell adhesion. Different abundance of disulfide isomerases caused different stoichiometric balance patterns between disulfide isomerases -integration interactions and therefore, induced different binding affinity, resulting in differential biological functions and regulatory effects (Swiatkowska et al., 2008). Therefore, stoichiometric balance is quite significant for PPIs.

Secondly, in addition to such PPI participants, the interactions between LamB and Odpq as another two effective proteins have also been influenced by the abundance of each protein and such abundance induced influences may further affect their potential biological functions, the antibiotic resistance in chlortetracycline-resistant Escherichia coli strain (Lin et al., 2014). Different abundance of such two participants may have totally opposite biological effects on such interactions: the interactions of lower concentration may improve the antibiotic sensitivity of E. coli, while the interactions at high concentration on the contrary directly induce the chlortetracycline-resistance. Therefore, the abundance of participants may be quite essential for PPIs. Similarly, another two features in the optimal feature list named as “Ce_CRF_wan_60_1209_poisson” and “Hs_helaC_mar_SGF_poisson” also contribute to the description of stoichiometric balance and protein abundance, validating their effective roles in the identification of actual PPIs.

Apart from such stoichiometric balance and protein abundance associated features, the following ten features can be further divided into two groups describing the molecular weight (“Ce_CRF_wan_60_1209_wcc,” “Ce_BNF_wan_60_1209_wcc”) and charge distribution (“Ce_CRF_wan_60_1209_pq_euc,” “Ce_BNF_wan_60_1209_pq_euc,” “Ce_beadsflow_1206_pq_euc,” “Ce_1111_pq_euc,” “Ce_beadsL_1206_pq_euc,” “Ce_6mg_1203_pq_euc”) of related proteins, respectively. The features that possibly affect the PPIs might be the molecular weight and the charge distribution of each PPI participant. These features have been validated by recent publications.

For instance, a study on SG2NA protein variants confirmed that the molecular weight and structure of such protein may directly affect its binding affinity against its ligands (Mitterhuber, 2008; Soni et al., 2014; Pinton et al., 2015). Therefore, molecular weight induced by different amino acid substitution may affect PPIs. The associations among different proteins were reported to be possibly strongly affected by long-range electrostatic interactions, and similar proteins with different surface charges may have different interaction patterns (Twomey et al., 2013; Raut and Kalonia, 2015). Therefore, the charge distribution of PPI participants affected the interactions between proteins.

### Analysis of Optimal PPI Rules

Based on the detailed parameter that corresponds to each optimal PPI feature extracted from the three datasets, the relatively quantitative rules to recognize potential PPIs were inferred (Table 3). The features that describe sensitivity gain factor confirmed that the PPI features and their parameters extracted from different datasets should be comparable, and the detailed analysis of each optimal PPI rule could be derived in the following discussions.

The literature confirmed rules with proper parameters may contribute to identifying potential PPIs and such predicted rules may act as reference for the prediction and screening of novel PPIs. In terms of the detailed quantitative features, two specific parameters, namely, “neg_ln_pval” and “hein_neg_ln_pval,” were identified in Rule1-Rule5. High relative (parameter) value of such two features indicate the interaction may actually happen. Although the detailed parameter (threshold) cannot be validated through wet-experiments at present, proper stoichiometric balance and protein abundance indicated by the parameters were discussed previously and already confirmed to promote the PPIs according to recent publications (Vinayagam et al., 2011; Fairweather et al., 2015). These rules could also be grouped in accordance with their new insights into the detailed biological mechanisms:

Apart from such two features, another two features have also been screened out to contribute to the quantitative identification of actual PPIs: “Hs_G166_1104_pq_euc” (used in Rule2-Rule5) and “pair_count” (used in Rule3-Rule5). In all the top rules apart from the first one which only involves “neg_ln_pval” and “hein_neg_ln_pval” as we have mentioned above, the value of “Hs_G166_1104_pq_euc” turns out to be lower than zero according to our quantitative rules.

According to the analyses above, such parameter contributes to the description of the charge distribution of certain PPI participants. Although no accurate description of such parameter, it has been confirmed that the higher the value is, the lower surface charging the participants of potential PPIs carries. Considering that it has been reported that charge interactions play an irreplaceable role for actual PPIs, therefore, potential interactions with such parameter lower than zero may not be actual PPIs. As for another parameter named as “pair_count,” in Rule3-Rule5, such parameter has a value >2, 3, and 3. It has been reported that the higher the value of such parameter may be, the less possible such interaction may actual happens (Hein et al., 2015). Therefore, interactions breaking such top five rules turns out to be actual PPIs, corresponding with our analyses above.

## Conclusion

Protein is the basic molecule of life. Through protein-protein interactions, complex biological processes are carried out. Predict PPI is a fundamental problem in bioinformatics. In this study, we encoded protein with various physical and chemical features, such as stoichiometric balance, protein abundance, molecular weight, and charge distribution. Then with advanced feature selection methods, we identified the key factors affecting PPIs and built a quantitative decision-rule system to evaluate the potential of PPIs under real conditions. Our results provided novel insights of the molecular mechanisms of PPIs. The model can be extended to explore other molecular interaction questions. The main datasets and codes can be downloaded at https://github.com/xypan1232/Mass-PPI.

## Data Availability Statement

Publicly available datasets were analyzed in this study. This data can be found here: the datasets for this study can be found in http://proteincomplexes.org/download.

## Author Contributions

TH and Y-DC designed the study. XP, KF, and LC performed the experiments. TZ and Y-HZ analyzed the results. XP and TZ wrote the manuscript. All authors contributed to the research and reviewed the manuscript.

## Conflict of Interest

The authors declare that the research was conducted in the absence of any commercial or financial relationships that could be construed as a potential conflict of interest.
